# Corrigendum: Modeling flow in an *in vitro* anatomical cerebrovascular model with experimental validation

**DOI:** 10.3389/fmedt.2024.1533412

**Published:** 2025-01-03

**Authors:** Saurabh Bhardwaj, Brent A. Craven, Jacob E. Sever, Francesco Costanzo, Scott D. Simon, Keefe B. Manning

**Affiliations:** ^1^ Department of Biomedical Engineering, Pennsylvania State University, University Park, PA, United States; ^2^ Office of Science and Engineering Laboratories, Center for Devices and Radiological Health, U.S. Food and Drug Administration, Silver Spring, MD, United States; ^3^ Department of Engineering Science and Mechanics, Pennsylvania State University, University Park, PA, United States; ^4^ Department of Neurosurgery, Penn State Hershey Medical Center, Hershey, PA, United States; ^5^ Department of Surgery, Penn State Hershey Medical Center, Hershey, PA, United States

**Keywords:** cerebrovascular model, cerebral blood flow, image based modeling, acute ischemic stroke, fluid dynamics

A Corrigendum on Modeling flow in an ***in vitro*** **anatomical cerebrovascular model with experimental validation** By Bhardwaj S, Craven BA, Sever JE, Costanzo F, Simon SD, Manning KB. (2023). Front. Med. Technol. 5:1130201. doi: 10.3389/fmedt.2023.1130201

**Error in Figure/Table**

In the published article, there was an error in Figure 3b as published. The simulation was based on an incorrect scale and the values have been updated. The corrected Figure 3b and its caption appear below.

**Figure 3:**
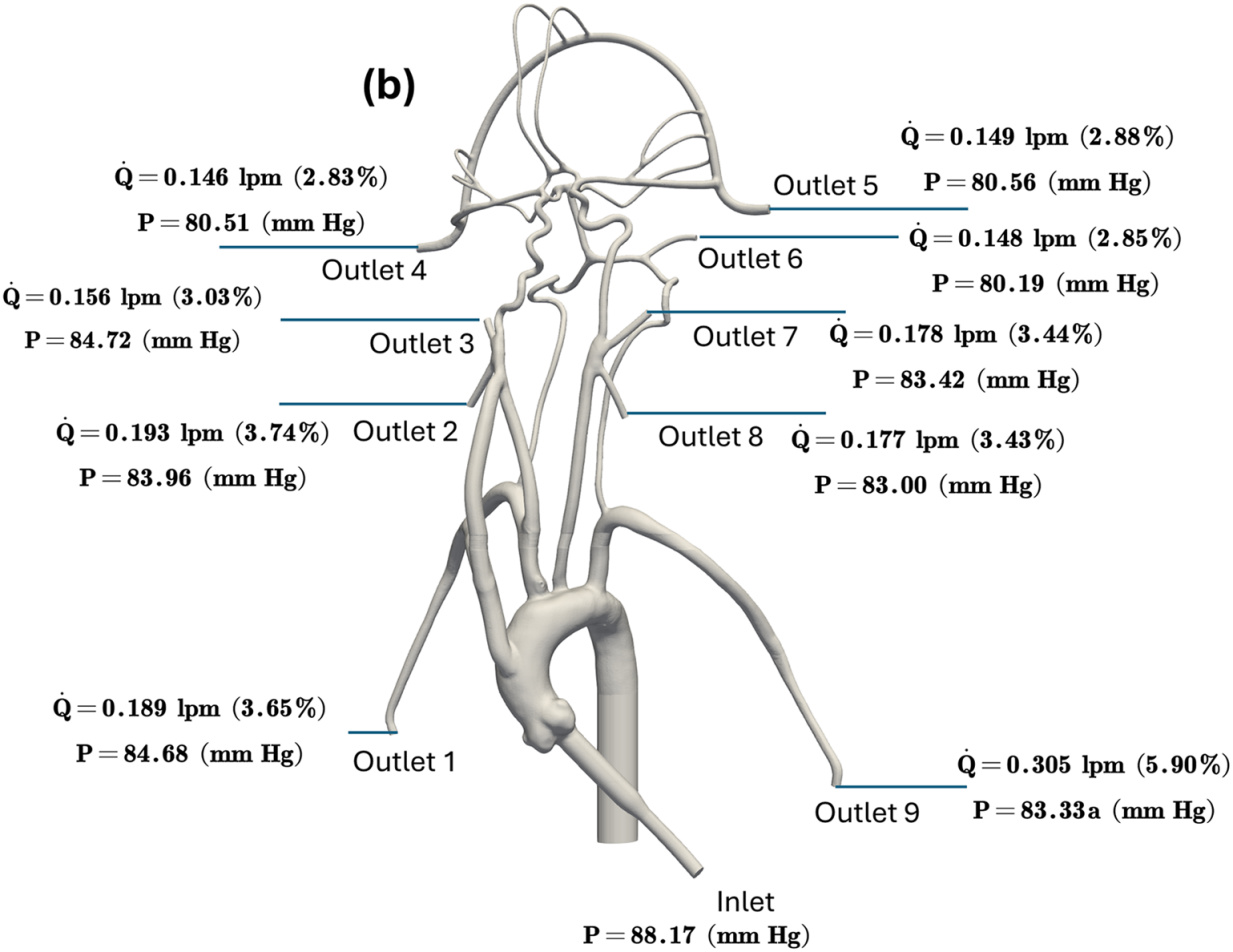
The mean volumetric flow rate and pressure values along with standard deviation at the inlet and all arterial outlets from **(a)** *in vitro* experiments and **(b)** CFD simulation for the normal condition. The percentage values shown in parentheses indicate the fraction of total inlet flow through each arterial outlet.

In the published article, there was an error in Figure 5 as published. The simulation was conducted using an incorrect scale and these values have been revised**.** The corrected Figure 5 and its caption appear below.

**Figure 5:**
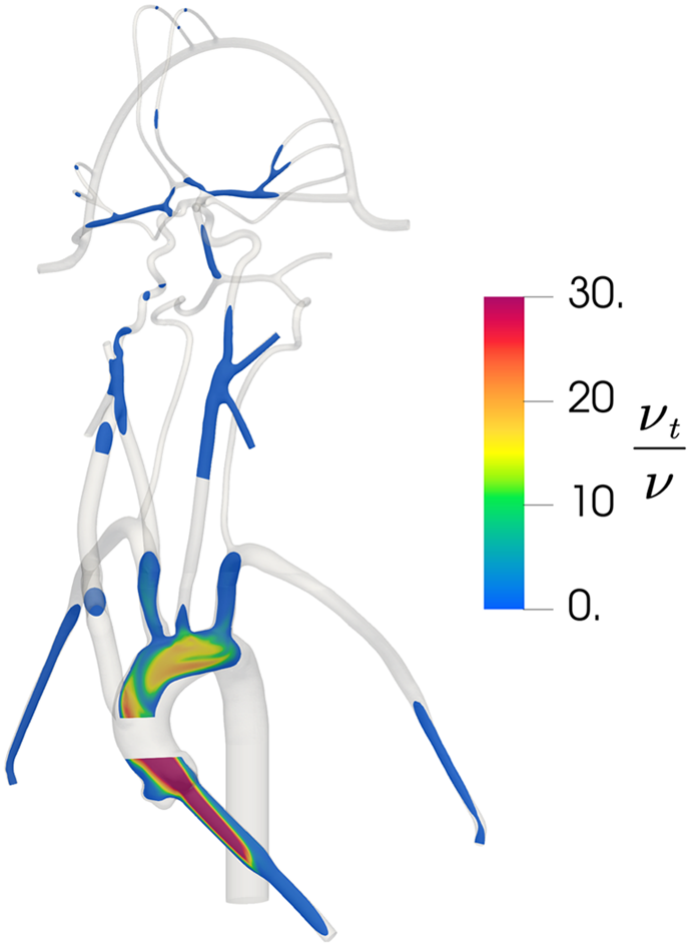
Contours of the distribution of the ratio of turbulent viscosity $\left(\nu_{t}\right)$ to molecular viscosity (ν) in the updated CFD model, illustrating regions of predicted turbulent flow where $\nu_{t}/\nu$ is large and regions of laminar flow where $\nu_{t}/\nu$ is small.

In the published article, there was an error in Figure 6 as published. The simulation was conducted using an incorrect scale and these values have been revised. The corrected Figure 6 and its caption appear below.

**Figure 6:**
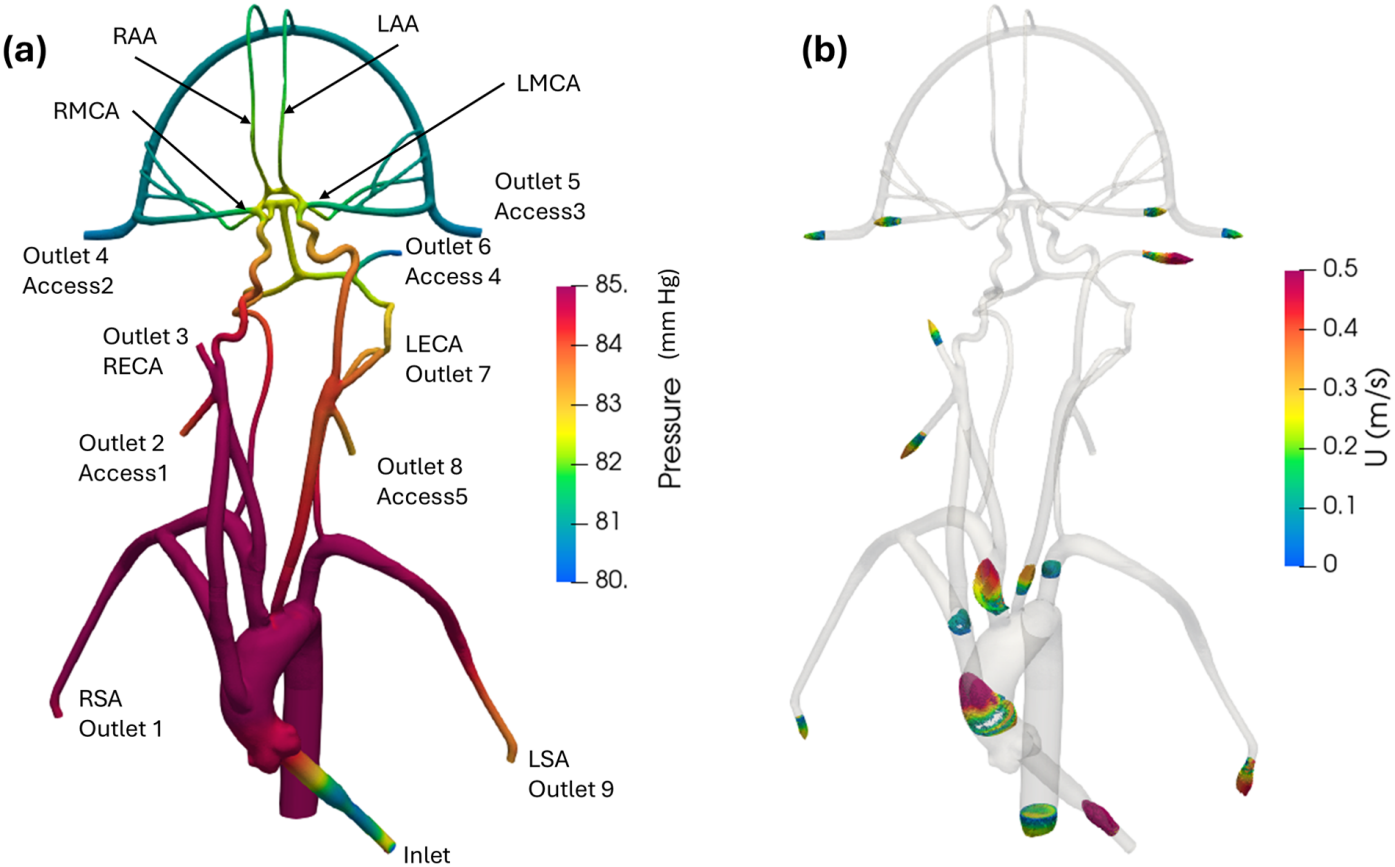
**(a)** Mean pressure contours and **(b)** profiles of mean velocity at various sections in the anatomical cerebrovascular model from the updated CFD for the normal condition.

In the published article, there was an error in Table 2 as published. The simulation was conducted using an incorrect scale and these values have been revised. The corrected Table 2 and its caption appear below.

**Table 2:** Mean pressure (mmHg) obtained from *in vitro* experiments and CFD at various inlet and outlets for the normal condition. The experimental values are reported as mean ± standard deviation (SD) from three experiments.

Location	*In vitro* pressure (mm Hg)	CFD pressure (mm Hg)	% Difference
Inlet	84.91 ± 0.9	88.17	3.83
Outlet 1 (RSA)	83.84 ± 0.67	84.68	1.00
Outlet 2 (Access1)	82.56 ± 0.32	83.96	1.70
Outlet 3 (RECA)	83.93 ± 0.37	84.72	0.94
Outlet 4 (Access2)	82.11 ± 1.5	80.51	1.95
Outlet 5 (Access3)	83.43 ± 1.8	80.56	3.44
Outlet 6 (Access4)	83.51 ± 1.27	80.19	3.98
Outlet 7 (LECA)	83.90 ± 1.3	83.42	0.57
Outlet 8 (Access5)	83.83 ± 0.44	83.00	0.99
Outlet 9 (LSA)	80.31 ± 1.04	83.33	3.76

In the published article, there was an error in Table 3 as published. The simulation was conducted using an incorrect scale and these values have been revised. The corrected Table 3 and its caption appear below.

**Table 3:** Mean pressure (mmHg) obtained from *in vitro* experiments and CFD at the inlet and outlets for the stroke condition. The experimental values are reported as mean ± standard deviation (SD) from three experiments.

Location	*In vitro* pressure (mm Hg)	CFD pressure (mm Hg)	% Difference
Inlet	84.25 ± 0.79	85.58	1.58
Outlet 1 (RSA)	83.04 ± 0.85	82.48	0.67
Outlet 2 (Access1)	81.01 ± 0.72	81.75	0.92
Outlet 3 (RECA)	82.02 ± 0.67	82.55	0.64
Outlet 4 (Access2)	–	–	–
Outlet 5 (Access3)	80.04 ± 1.2	79.31	0.91
Outlet 6 (Access4)	78.83 ± 1.7	78.66	0.22
Outlet 7 (LECA)	80.40 ± 1.19	81.46	1.31
Outlet 8 (Access5)	79.55 ± 0.96	81.02	1.85
Outlet 9 (LSA)	78.74 ± 1.56	81.19	3.11

The authors apologize for this error and state that this does not change the scientific conclusions of the article in any way. The original article has been updated.

